# Cognitive Impairment in Parkinson’s Disease: Epidemiology, Clinical Profile, Protective and Risk Factors

**DOI:** 10.3390/bs11050074

**Published:** 2021-05-13

**Authors:** Paulina Gonzalez-Latapi, Ece Bayram, Irene Litvan, Connie Marras

**Affiliations:** 1 Edmond J. Safra Program in Parkinson’s Disease and the Morton and Gloria Shulman Movement Disorders Clinic, Toronto Western Hospital, University Health Network, Toronto, ON M5T2S8, Canada; paulina.latapi@uhnresearch.ca; 2 Parkinson and Other Movement Disorders Center, Department of Neurosciences, University of California San Diego, La Jolla, CA 92093, USA; drecebayram@gmail.com (E.B.); ilitvan@ucsd.edu (I.L.)

**Keywords:** Parkinson’s disease, dementia, mild cognitive impairment, risk factors, clinical profile

## Abstract

Cognitive impairment is a common non-motor symptom in Parkinson’s Disease (PD) and an important source of patient disability and caregiver burden. The timing, profile and rate of cognitive decline varies widely among individuals with PD and can range from normal cognition to mild cognitive impairment (PD-MCI) and dementia (PDD). Beta-amyloid and tau brain accumulation, oxidative stress and neuroinflammation are reported risk factors for cognitive impairment. Traumatic brain injury and pesticide and tobacco exposure have also been described. Genetic risk factors including genes such as *COMT, APOE*, *MAPT* and *BDNF* may also play a role. Less is known about protective factors, although the Mediterranean diet and exercise may fall in this category. Nonetheless, there is conflicting evidence for most of the factors that have been studied. The use of inconsistent criteria and lack of comprehensive assessment in many studies are important methodological issues. Timing of exposure also plays a crucial role, although identification of the correct time window has been historically difficult in PD. Our understanding of the mechanism behind these factors, as well as the interactions between gene and environment as determinants of disease phenotype and the identification of modifiable risk factors will be paramount, as this will allow for potential interventions even in established PD.

## 1. Introduction

The classic view of Parkinson’s disease (PD) has been dominated by the key misconception that it is a neurodegenerative disorder that predominately affects dopamine-producing neurons of the substantia nigra, resulting in motor symptoms. Now, we understand that PD is a heterogeneous multisystem disorder comprising a wide range of motor and non-motor symptoms. These non-motor symptoms are frequent and become increasingly prevalent with advancing disease. 

Cognitive decline is among the most common non-motor symptoms. As one of the most severe and disabling non-motor symptoms of PD, cognitive impairment, particularly dementia, has been statistically associated with mortality [1]. In addition, cognitive impairment impairs social function, intensifies caregiver burden and the costs of disease-related medical care [2]. The pathophysiology of cognitive impairment in PD is complex and likely involves a disruption of multiple distinct neural networks occurring over time [3]. The timing, profile and rate of cognitive decline varies widely among individuals with PD. As such, it is important to better comprehend the clinical profile of cognitive impairment in PD as well as the risk factors for developing these cognitive changes. This understanding is crucial clinically for communicating prognosis and managing patients. A better understanding of these factors also allows for optimization of research efforts, particularly clinical trial design.

In this review, we will highlight the clinical profile associated with cognitive impairment in PD, including mild cognitive impairment (MCI) and dementia (PDD). We will also review the most recent evidence regarding risk and protective factors associated with cognitive changes in PDD. Given the morbidity associated with cognitive decline, preventative and modifying strategies would have great impact and may be found through knowledge of such factors. Finally, we highlight the gaps in the literature and future research directions. 

## 2. Clinical profile 

### 2.1. Cognitive Profile in Parkinson Disease

The cognitive profile in PD can range from normal cognition to MCI and eventually dementia. Therefore, patients can have normal scores on neuropsychological testing with and without subjective complaints (normal), impairments on neuropsychological testing without (MCI) or with a substantial impact on daily instrumental life activities (dementia). Clinical diagnosis of PD is primarily based on the presence of motor symptoms [4]. Nonetheless, the neurodegenerative process underlying PD starts long before the onset of the motor symptoms [5], and cognitive changes can occur during these earlier phases of neurodegeneration. Accordingly, several reports have shown increased PD risk in people with cognitive changes, and cognitive deficits are included in the prodromal PD criteria [6,7,8,9]. In longitudinal studies including individuals without PD at baseline who later developed PD during the follow-up period, subtle cognitive changes occurred within 7 to 9 years before the diagnosis of PD [10,11], and worse cognitive performance has been associated with a higher probability of prodromal PD [7,12]. Although the presence of dementia before or close to the onset of parkinsonism is more suggestive of a clinical diagnosis of dementia with Lewy bodies (DLB) [13], these reports show that cognitive deficits at the level of subjective complaints and/or MCI can occur before the onset of PD and have predictive value for PD development.

After the diagnosis of PD, patients can present with subjective complaints which may or may not be accompanied by objective changes [14,15]. Even if those patients do not have impairment on an objective neuropsychological measure, they should be monitored for cognitive decline as subjective complaints can predict the development of MCI over a two-year follow-up in newly diagnosed PD patients [14]. In terms of the cognitive profile in early PD, the majority of patients have a non-amnestic single-domain cognitive decline with impairments in visuospatial functioning, attention, or executive functioning [16,17,18]. Additionally, impairments on tests assessing language and visuospatial functioning have a higher sensitivity for predicting dementia [19,20]. However, patients do not always have single-domain cognitive decline, and decline can be present across multiple and/or all those cognitive domains [17]. To explain this heterogeneity for the affected cognitive domains in PD, the “dual syndrome hypothesis” has been proposed [21]. This hypothesis suggests that in PD patients with more fronto-striatal network dysfunction, modulated by dopamine, attention/working memory and executive functions are predominantly affected, whereas in patients with more posterior cortical degeneration, memory, language, and visuospatial functioning are predominantly affected related to greater cholinergic loss. Therefore, comprehensive evaluation of individual cognitive domains is crucial for estimating the underlying pathophysiology and subsequently lead to development of effective treatments. Admittedly, the cognitive profile can be hard to discern if the patient’s cognitive decline is too advanced with severe impairment across all domains at the time of testing. Sex-specific patterns for PD-associated cognitive alterations have also been described, with verbal fluency and lack of facial emotions being more prevalent in males and a reduction in visuospatial cognition appearing more frequently in females [22]. 

As the neurodegenerative progression continues in PD, cognitive changes also advance; the frequency of patients with MCI increases [23], and those with MCI are at a higher risk for the development of dementia [24]. Although dementia may seem almost inevitable after 20 years following the diagnosis of PD, with dementia prevalence reaching 80% [25], the rate of progression to dementia is highly variable. The additional high variability for the affected cognitive domains and the severity of the cognitive decline in PD makes it challenging to make predictions about the progression and underlying pathologies. Although there are currently no pharmacological treatments to delay or prevent cognitive decline in PD, from a research perspective it is important to be able to detect the patients at risk before the onset of severe cognitive decline and to monitor the progression to determine the rate of decline. Detecting such patients and progression rates will enable the development of effective strategies to prevent or delay progression to dementia in PD. 

### 2.2. Diagnostic Criteria

To promote uniformity amongst those who work on cognition in PD, clinical criteria for stages of cognitive decline (MCI and dementia) have been previously defined by expert panels for use in clinical and research settings [26,27]. These stages are based on a combination of patient and caregiver reports, clinician observations, and neuropsychological assessments of the patients. The diagnosis of PD-MCI is based on (1) the individual having a diagnosis of PD, (2) a gradual cognitive decline reported by the patient, informant, or the clinician, (3) cognitive decline on comprehensive neuropsychological testing or a scale of global cognitive abilities validated in PD and (4) cognitive decline not sufficient to interfere significantly with functional independence [27]. PD-MCI represents the prodromal stage of PD dementia (PDD) and can provide a window to prevent or delay the progression to PDD. The PD-MCI diagnostic criteria have two levels with Level I implying brief assessment and Level II implying a more comprehensive assessment with at least two tests for each of the five cognitive domains (attention, executive function, visuospatial functioning, language, memory) [27]. If Level II testing can be performed, the patient needs to show impairments on two tests in one cognitive domain or one impaired test in two different cognitive domains. Impairment is defined by performance 1-2 standard deviations (SDs) below appropriate norms, decline from prior neuropsychological testing, and decline from estimated premorbid levels [27]. Both Level I and II criteria are valid predictors of PDD [24,28], however, detailed neuropsychological evaluations can be more sensitive to detect cognitive decline [29]. Given the heterogeneous nature of the cognitive decline in PD, detailed neuropsychological evaluations assessing various cognitive domains can provide a more comprehensive view of the cognitive state. 

The diagnosis of PDD is based on (1) the individual having a diagnosis of PD, (2) a slowly progressive cognitive decline that developed after an established PD diagnosis, and (3) impairment in more than one cognitive domain, which represents a decline from premorbid level and is severe enough to impair instrumental daily life activities [26]. Behavioral symptoms including hallucinations, delusions, apathy, depression, anxiety, personality changes, and excessive daytime sleepiness frequently co-occur with PDD but are not required for the diagnosis of PDD [26]. Nevertheless, these behavioral symptoms impact the quality of life of both the patient and the caregiver [30] and need to be addressed by the clinician. 

For both PD-MCI and PDD, the presence of other conditions which may explain the cognitive impairment (e.g., delirium, stroke, trauma, metabolic abnormalities) and other PD-related comorbidities (e.g., motor impairment, severe depression, anxiety, excessive daytime sleepiness, psychosis) that may significantly influence neuropsychological testing need to be taken into account [26,27]. As global cognitive screening tools, The Montreal Cognitive Assessment, the Mattis Dementia Rating Scale Second Edition, and the Parkinson’s Disease-Cognitive Rating Scale were recommended for use in PD by the MDS Rating Scales Review Committee [31]. These three scales adequately represent relevant cognitive domains and were found to be reliable, valid and sensitive to change in PD. In terms of detailed neuropsychological testing, there is no optimal battery to detect cognitive changes in PD for the time being. Different neuropsychological tests and norms are used across centers and for different research studies and for clinical assessment. However, preliminary work has been performed to guide future efforts to establish an efficient neuropsychological battery. Out of 19 MDS-recommended scales representing five cognitive domains, the two best performing tests for each cognitive domain were determined (Table 1) with 2 SDs below norms on these tests suggested to be highly sensitive and specific for PD-MCI diagnosis [32]. However, a multi-site study by the MDS Study Group for Validation of MCI in PD, which included 2908 PD patients and 1247 healthy controls, did not provide strong support for this threshold [33]. Different sites were using different normative scores for neuropsychological tests, which led to a high variability in cognitive performance across study sites. This between-site variability was reduced when comparisons involved healthy controls that were matched with the patients for education, language, test version, test procedures, and source population within the studies. The study group outlined the need to unify existing guidelines for test procedures, including matched healthy controls, and developing more extensive normative data for tests to account for age, education, sex, ethnicity and race to allow comparability of findings in research. In a clinical setting, repeated neuropsychological testing to determine the change over time and consideration of various factors while interpreting the results including the patient’s age, sex, education, motor and non-motor symptom severity, as well as functioning in instrumental daily life activities will help reach a more reliable conclusion about their cognitive status.

### 2.3. Prevalence and Incidence of Cognitive Impairment in Sporadic and Genetic Forms of PD

The reported prevalence of PD-MCI varies widely, which has been attributed to the use of differing criteria in many studies. Studies using the Movement Disorder Society Task Force Level II criteria, enrolling early stage (H&Y Stage 2) and *de novo* PD (within 2 years of symptom onset), report prevalence varying from 20% to 41% [23,34]. A population-based cohort of early-stage PD found that in those with normal cognition at baseline, the cumulative incidence of PD-MCI was 9.9% after 1 year and 28.9% after five years of follow-up [34]. A recent meta-analysis reported pooled PD-MCI prevalence to be 40% on a total sample of 7053 PD patients. As for type of PD-MCI, prevalence of multiple-domain was 31% [35]. Interestingly, only disease progression (assessed by H&Y scale) significantly moderated the prevalence rate of PD-MCI. Other variables including older age, longer disease duration, depression and apathy did not moderate prevalence estimates but were significantly different between PD with and without MCI. Interestingly, lower education levels also characterized PD-MCI [35]. 

About 10% of PD patients develop dementia every year, which is four to six times higher than that of non-PD patients [36], and the life-long prevalence of PDD is almost 80% [25,28,37,38]. Patients with PD-MCI are also more likely to eventually develop dementia [25] and to develop it earlier [39,40] than PD patients without cognitive impairment. 

Subjective cognitive complaints are also associated with progression to dementia. A recent study showed that in patients with PD who expressed subjective cognitive complaints at baseline, a third had progressed to PD-MCI and PDD within 7.5 years [41,42].

Most cases of PD are sporadic, although a significant minority have strong genetic determinants (termed ‘monogenic PD’) and a proportion of individuals carry one or more genetic variants known to increase PD risk. Clinically, there are differences between the sporadic and monogenic forms of PD, and this includes the risk of cognitive decline. Some of the most studied genes include alpha-synuclein (*SNCA*), leucine-rich repeat kinase 2 (*LRRK2)*, as well as the glucocerebrosidase (*GBA*) gene. Importantly, genetic risk variants may also have an influence on the incidence and rate of progression of cognitive impairment in sporadic PD and this topic is discussed later in our review. 

*SNCA* mutations cause autosomal-dominant PD and are associated with severe effects on cognition. A recent review using data from the MDSGene database (www.mdsgene.org) concluded that 70% of *SNCA* carriers were affected by cognitive decline. *SNCA* mutation carriers also show a shorter time from emergence of motor symptoms to the onset of dementia and a considerably younger age at onset of dementia than sporadic PD. Even more, there appears to be a dosage effect, with 88% of *SNCA* triplication carriers showing cognitive changes, compared to 68% of duplication carriers [43]. Aside from disease-causing mutations associated with autosomal dominant inheritance patterns, a variant in intron 4 of *SNCA* has also been associated with a higher risk of PDD [44]. This raises the possibility that *SNCA*-related mechanisms are associated with cognition in sporadic PD, warranting additional studies in larger and also more ethnically diverse cohorts. 

Mutations in the *GBA* gene are reported to be associated with faster rates of motor and cognitive progression compared with sporadic PD. Zhang et al (2015) showed that *GBA* mutations were associated with a 3.2-fold increased risk of dementia or cognitive impairment [45]. More recently, Creese et al (2017) confirmed these findings, showing a 2.4-fold increase in risk of cognitive impairment in PD subjects with *GBA* mutations [46]. Furthermore, *GBA* gene mutations are also associated with more rapid cognitive decline [47,48,49]. This has been described for both “severe” (L444P) and “mild” (N370S) *GBA* mutations [47,49,50]. Nonetheless, data from the Parkinson’s Progression Markers Initiative (PPMI) shows no difference in MoCA score or detailed neurocognitive battery between *GBA* PD and sporadic PD patients (*n* = 80 and 361, respectively) [51]; this may be due to shorter disease duration (3 years) compared to other cohorts. These data have interesting implications for the timeline of the dissociation of this phenotype from sporadic PD and suggest that the cognitive differences emerge predominantly at later disease stages. Since the PPMI analysis was cross-sectional, longitudinal data will be useful for defining the slope of progression and comparing it with sporadic PD. 

Mutations in *LRRK2* are the most common cause of late-onset autosomal-dominant PD, with *LRRK2* Gly2019Ser as the predominant variant. *LRRK2* G2019S mutations carriers are reported to have less non-motor disabilities and a slower rate of PD progression, compared to sporadic PD [52]. Recently, Simuni et al (2020) described no meaningful difference in cognitive performance between *LRRK2* and sporadic PD subjects [51]. This is consistent with previous reports from other studies [52,53]. 

The effect of less frequent variants is more difficult to ascertain due to the small number of cases available for study, as well as inconsistent use of diagnostic criteria for PD-MCI and PDD. A systematic review summarizing cognitive and psychiatric manifestations of genetically determined PD, found that patients with *PINK1* (*n* = 24) mutations had the highest incidence of cognitive decline, followed by *SNCA* (*n* = 151) and *DJ1* (*n* = 8) mutation carriers. *VPS35* (*n* = 24) and *LRRK2* (*n* = 1625) had the lowest rates of cognitive impairment [54]. An earlier review that used MDSGene data and included a total of 1127 patients with causative *Parkin* (*n* = 958)*, PINK1* (*n* = 139) or *DJ1* (*n* = 30) mutations suggests that cognitive impairment was present in only a small percentage of subjects with *PINK1* associated PD [55]. Cognitive changes also appear to be rare in *Parkin* mutation carriers [56]. 

Further work in longitudinal cohorts of genetic PD in both pre-symptomatic and symptomatic individuals is needed to better elucidate the nature of the observed associations. Additionally, there are no available studies that specifically examine the association between neural network dysfunction, neuropathological findings and domain impairment in genetic PD. 

## 3. Risk Factors for Cognitive Decline in Parkinson Disease

### 3.1. Sex

Male sex has been associated with a higher risk of cognitive impairment. The Oxford Parkinson Disease Centre cohort included PD patients within 3 years of diagnosis. Most indices of cognitive performance, including MoCA and phonemic and semantic fluency, were significantly worse in males compared to females. There was no difference in MMSE scores. Males also had a greater rate of REM behavior disorder (RBD) and orthostatic hypotension [57]. Similar results were reported in an analysis of the baseline data from the National Institutes of Health Exploratory Trials in Parkinson’s Disease (NET-PD); where Augustine et al (2015) examined differences in the clinical features and disease severity of male and females with early treated (PD). Interestingly, while there were no differences in age at PD onset, diagnosis or motor symptoms, women demonstrated better performance compared to men on SCOPA-COG and Symbol Digit Modality measures [58]. In contrast, Gao et al (2015) reported lower MoCA scores in female patients among a cohort of 311 PD patients attended at the Department of Neurology in Guangdong General Hospital. MMSE scores did not show a significant difference between males and females [59]. In contrast to other cohorts, education level was significantly lower in female patients and may account for these results.

There may also be domain-specific sex difference patterns, with males with PD showing worse performance on verbal memory tests and better visuospatial abilities, compared to females with PD [60]. A similar pattern has been described in healthy elderly subjects [61] and patients with Alzheimer disease (AD) [62]. 

The mechanism linking male sex and cognitive performance has not been elucidated. As further explored in the following sections, RBD and orthostatic hypotension have also been associated with worse cognitive performance. These symptoms were also more frequent in males in the above cohort and may be contributing to worse cognitive performance. Finally, most cohorts have included early-onset PD subjects, so it remains to be seen if this trend continues in late disease stages. 

### 3.2. Comorbidities 

#### 3.2.1. Alzheimer Disease Pathology

Evidence from postmortem studies indicates that limbic and cortical Lewy pathology correlates with dementia in PD. Higher total burden of α-synuclein was associated with faster decline in cognitive performance in de novo patients [63] and advanced analyses show that PDD subjects have more oligomeric forms of α-synuclein [64,65]. However, there is increasing recognition that Lewy pathology is not the only determinant of cortical dysfunction and cognitive decline in PD. In addition, there is strong evidence that amyloid and tau pathology also contribute to cognitive changes [66,67]. A recent systematic review showed that one-third of PDD cases also fulfilled pathological criteria for AD, with moderate to severe tau pathology in one-third and moderate to severe amyloid-β pathology in half [68]. Cerebrospinal fluid analysis shows lower levels of amyloid-β in PDD compared to healthy controls and individuals with PD without dementia; lower levels were also associated with progression to dementia in non-demented patients [64,69,70,71]. Data for total tau and phosphorylated tau (p-tau) are less consistent [69,72,73]. In PD-MCI, amyloid-β has been found to be either equal to or less than that in PD patients with normal cognition and p-tau was comparable [74,75]. The relative contribution of amyloid-β and tau pathology versus α-synuclein to cognitive dysfunction is more difficult to define. The combination of α-synuclein, amyloid- ß and tau pathology seem to have an additive effect on cognitive changes in PD [76,77,78,79,80,81,82]. Compared to amyloid-β, tau pathology seems to have a closer relationship with cognitive status in PD, particularly when α-synuclein deposition is low [83]. These findings may have implications for clinical trials of disease-modifying immunotherapies, particularly those targeting tau deposition. 

Other pathologies may also contribute to dementia in PD. Cerebral amyloid angiopathy (CAA) was significantly more common in PDD compared with PD without dementia and correlated with coexistent AD pathology [84]; although another study suggested a stronger association between CAA and dementia with Lewy bodies [85]. TDP-43 and argyrophilic grain disease are rare pathological findings and do not appear to be associated with dementia in PD [86,87]. 

#### 3.2.2. Sleep and Mood Disorders

Sleep disorders are a common complication of PD and may predict cognitive changes. Analysis of participants in PPMI show that even at early stages (H&Y stage one and two) PD patients with possible REM behavior disorder (pRBD) were more likely to have MCI. On follow-up, PD patients with pRBD experienced a decline of 0.34 points per year on the Montreal Cognitive Assessment (MoCA) more than in those without pRBD, which is equivalent to the effect that a 7-year increase in age would have on the MoCA score [88]. The odds of MCI at any point during follow-up was significantly greater among those with pRBD at baseline [88]. Importantly, there was no independent association between insomnia and rate of cognitive decline, suggesting the mechanisms extend beyond sleep quality. A subsequent study with longer follow-up data (8 years), corroborated that pRBD was associated with faster progression of cognitive impairment in PD and also showed a significant association between cognitive decline and excessive daytime sleepiness [89]. A separate cohort study highlighted the adverse prognostic implications of RBD in PD; besides a higher prevalence of MCI at baseline patients with RBD also had a 15% risk of dementia at 2 years and 45% at 4 years [90].

Mood disorders have also been associated with faster progression to cognitive impairment in early PD. A recent study in a large cohort followed for up to 8 years provides evidence for a significant association between early depression and anxiety and faster decline in MoCA scores over time [89]. However, a systematic review by Guo et al (2019) did not find a significant relative risk for cognitive impairment and depression [91]. 

#### 3.2.3. Cardiovascular Risk Factors 

In the general population, some modifiable risk factors, including hypertension, diabetes, obesity and hypercholesterolemia have been associated with cognitive impairment and dementia [92]. In PD, some studies have associated these factors with worse cognitive performance as well. An analysis of the PPMI cohort found that a vascular risk score made up of the combination of hypertension, diabetes mellitus and body mass index was associated with the presence of white matter hyperintensities for total brain frontal and temporal regions [93]. White matter hyperintensities reflect white matter tissue ischemia pathologically and are a strong radiological predictor of cognitive decline in the general population [94,95]. In this same cohort, the annual rate of decrease in global cognition was greater in those with a higher vascular risk score [93]. An analysis of the De Novo Parkinson (DeNoPa) cohort showed that elevated fasting glucose, hemoglobin A1c and elevated C-reactive protein were identified as independent factors associated with yearly Mini-Mental State Examination score decline in PD participants, compared to healthy controls [96]. 

A recent study, also using the PPMI cohort, confirmed that higher BMI at baseline was associated with faster cognitive decline in early PD [89]. Nonetheless, weight loss over the course of PD has also been linked to faster cognitive decline [97,98] and overweight and obese PD patients have been described to have slower cognitive decline in language, memory and global cognition after 8 years [98]. As such, the relationship between weight loss and cognitive decline in PD appears to be complex and needs to be clarified by prospective studies. 

Blood pressure fluctuations have also been associated with cognitive impairment. Specifically, orthostatic hypotension (OH) has been associated with dementia risk in PD, particularly a decrease in diastolic blood pressure [99]. Repeated episodes of hypoperfusion and altered regional patterns in supine cerebral blood flow are likely mechanisms contributing to cognitive changes in PD [100]. Patients with PD and OH may also experience supine hypertension, either as a result of autonomic dysfunction due to underlying disease, or as an effect of the medications used to treat OH. Supine hypertension may also increase the risk of cerebral ischemic lesions, which as above, can contribute to cognitive decline in PD [93]. 

Despite the above data and proposed vascular mechanisms, autopsy studies suggest a minor role for ischemic cerebrovascular involvement in PDD. In one study, clinicopathological data from 97 PD subjects showed ischemic cardiovascular disease lesions (iCVD) in 20 subjects, including lacunar and cortical lesions, with no significant association between ischemic lesions and history of dementia [101]. Another autopsy study that included both Lewy Body Dementia and PD subjects, showed an inverse correlation between Lewy pathology and atherosclerosis, small vessel disease or ischemic lesions [102], suggesting that patients with a higher Lewy pathology burden were less likely to show severe cerebrovascular pathology or a history of stroke. 

Diabetes mellitus (DM) is another common vascular risk factor linked to both subtle and severe forms of cognitive dysfunction. DM has also been associated with cognitive changes in PD. Importantly, genetic [103,104] and epidemiologic studies [105,106] suggest that there are shared molecular pathways between DM and PD. Bosco et al (2012) showed that PDD patients had a higher prevalence of abnormal glucose metabolism and insulin resistance compared to nondemented PD [107]. This was further confirmed in a study comparing PD subjects with and without DM, which showed that DM was a predictor for cognitive decline with a hazard ratio of 4.6% [108]. Interestingly, in a recent cohort of PD patients from South Korea, DM was not associated with worse mean MMSE scores, but detailed neuropsychological assessment did show a significant association with worse scores in attention, working memory and frontal executive dysfunction [109]. The exact pathophysiological mechanism by which cognitive changes associated with DM remain to be elucidated but seem to extend beyond vascular disease. 

#### 3.2.4. Uric Acid

Urate is the most important natural antioxidant in humans that scavenges hydroxyl radicals, singlet oxygen and oxo-heme oxidants and has been inversely associated with PD risk [110,111,112]. Several studies have investigated the interaction between uric acid (UA) and cognitive changes in PD. A meta-analysis showed a positive correlation between serum UA and MMSE [113]. UA is reduced in the CSF of PDD patients [114] and in non-demented patients, low plasma UA concentrations were associated with a worse outcome in global cognition, attention and memory [115,116,117].

### 3.3. Inflammation and Oxidative Stress

Neuroinflammation has been implicated as an important factor for cognitive decline in PD. Microglial activation may drive progressive damage of dopaminergic neurons through the release of pro-inflammatory cytokines such as tumor necrosis factor-α (TNF-α), interleukin-6 (IL-6), interleukin 1β (IL-1β) and interferon-γ (INF-γ) [118]. Microglial activation was associated with decreased glucose metabolism (a marker of neuronal damage) in several brain regions, including the frontal lobe, in PD patients with dementia [119]. Inflammatory markers, including C-reactive protein [120] and IL-6 [75] and oxidative stress markers nitric oxide and hydroxyl radical have been found to be elevated in the CSF of patients with PD-MCI and correlate with lower MoCA scores [75]. Mitochondrial dysfunction can lead to an increase in oxidative stress as well as glial and astrocyte dysfunction, contributing to an abnormal inflammatory response [121,122,123]. In PDD, deficiency in mitochondrial complex 1 activity and reduced mitochondrial DNA levels in the prefrontal cortex, compared to healthy controls, have been described [124]. Neither was significantly reduced in PD-normal cognition patients, suggesting this could be a hallmark of dementia in patients with PD. However, further studies to replicate these findings and further elucidate the specific contributions of mitochondrial dysfunction and oxidative stress vs inflammation to PD-MCI and PDD are needed. Importantly, environmental exposures, including some associated with PD risk and cognitive impairment in PD, can contribute to oxidative stress. These possible contributing factors are explored in the next section on environmental risk factors for cognitive impairment in PD. 

### 3.4. Environmental Risk Factors 

#### 3.4.1. Traumatic Brain Injury

Traumatic brain injury (TBI) is a leading cause of disability worldwide. TBI often results in debilitating long-term cognitive impairment, likely as a result of secondary events that follow primary impact, such as neuronal cell death, oxidative stress, brain edema, blood-brain barrier breakdown and inflammation [125]. The association between PD and TBI has been widely debated due to conflicting studies [126,127,128,129]; however it appears that parkinsonism may be particularly associated with repeated trauma [130]. Yet, few studies have explored the association between TBI and the progression of cognitive impairment in PD. Schiehser et al (2016) showed that individuals with PD and a history of mild to moderate TBI evidenced greater cognitive decline (measured using the Mattis Dementia Rating Scale) over time compared to those without a history of TBI, despite similar disease severity, mood profile and changes in levodopa equivalent dose. These cognitive changes were mainly in the areas of executive function and memory, which are associated with brain regions most susceptible to head injury [131]. Further longitudinal studies with a comprehensive cognitive profile are needed to better elucidate this possible association. 

#### 3.4.2. Pesticide Exposure 

Pesticides have been repeatedly identified as a risk factor for PD [132] through mechanisms that may include the induction of oxidative stress, α-synuclein aggregation and microglial activation [133]. It has been hypothesized that pesticide exposure may also be associated with more rapid progression of PD, although studies specifically exploring cognitive changes are lacking. Analysis of the Fox Insight cohort showed a higher likelihood of cognitive impairment in PD patients with a *GBA* risk variant who also had an occupational exposure to pesticides. This association was not found in PD subjects with *LRRK2* pathogenic variants [134], suggesting that environmental exposures may have distinct effects on cognition in different genotypes. 

Other agents that influence oxidative stress may also play a role in cognitive decline in PD. 

#### 3.4.3. Tobacco 

Use of tobacco products falls is an important lifestyle choice that can impact cognition. Nicotine has been suggested to have a protective effect against PD in prior studies [135,136], and tobacco smoking is well-established as having an inverse association with PD risk [137]. However, the role of nicotine on cognition remains controversial and most studies in PD have focused on tobacco smoking rather than nicotine itself. A meta-analysis of prospective cohort studies reports that tobacco smoking increased the risk of cognitive decline in PD [91]. The Parkinson’s Environment and Gene study in central California enrolled 360 PD patients within 3 years of diagnosis who had a history of smoking. Compared to never smokers, current cigarette smoking was associated with a three-times higher risk of cognitive decline, defined as a four-point change on the MMSE. This association was not found in former smokers and there was no association with the number of pack-years [138], suggesting effects predominantly related to ongoing exposure. In a different study which included 139 PD patients, ever smokers had significantly worse MMSE scores compared to never smokers and the number of smoking pack-years was a significant predictor of MMSE scores, along with age and education [139]. More recently, the Parkinson disease cognitive impairment study (PACOS) explored the association of vascular risk factors and the occurrence of PD-MCI and its progression to PDD. This study included 139 non-demented PD subjects who underwent a comprehensive neuropsychological assessment; on follow-up there was no association between tobacco smoking and either PD-MCI or PDD [140]. The majority of studies suggest tobacco smoking as a risk factor for cognitive decline. It may be that, while nicotine counterbalances the cholinergic deficits involved in cognitive decline, increased oxidative-stress and cerebrovascular damage likely exceed any potential benefit that nicotine may provide. 

### 3.5. Genetic Risk Factors

In addition to rare genetic variants associated with monogenic forms of PD, there is interest in the effect that common variants in other genes may have on PD and cognition. Genes in this group include those involved in dopamine metabolism, brain development as well as those that have been associated with other dementias, such as Alzheimer Disease and frontotemporal dementia. Results have been varied and often contradictory.

Apolipoprotein E (*APOE*) genotype has a well-established association with susceptibility to Alzheimer Disease, with the *APOE ε4* allele associated with both AD risk and lower age at disease onset. The *APOE ε4* allele is also a plausible candidate for influencing PDD. A study combining cognitive testing with functional neuroimaging linked *APOE ε4* to impaired performance and abnormal activity in the temporo-parietal network in newly diagnosed PD [141]. A prospective population-based cohort also showed that *APOE ε4* genotype was associated with global cognitive decline [142]. In contrast, an incident cohort of 107 PD patients from the UK showed no significant association between the *APOE ε4* isoform and cognitive decline after 5 years [143], using the Mini-Mental State Examination as the sole primary outcome to assess cognitive function in PD. Thus, the role of *APOE* genotype in cognitive impairment in PD remains to be understood. 

Microtubule-associated protein tau (MAPT) is involved in microtubule assembly and stabilization. The *MAPT* H1/H1 genotype has been firmly established as a risk factor for tauopathies and is associated with PD risk. Association with cognitive dysfunction in PD was first reported in the Cambridgeshire Parkinson’s Incidence from GP to Neurologist (CamPaIGN) cohort. The 10-year follow up continued to support an association with H1/H1 genotype and progression to dementia, although the magnitude of the effect lessened with increased disease duration [144]. This association was replicated in a large case-control study where the H1 haplotype was overrepresented in PD patients, with a stronger association in PDD patients [145]. Nonetheless, this association was not found in two recent studies, which included patients with a longer disease duration [146,147,148]. The lack of association in cohorts with longer disease duration suggests that the predominant effect may be to accelerate cognitive decline in the early years of the disease. 

The *COMT* gene harbors a common polymorphism, Val158Met, which alters enzyme activity, with a 40% lower enzyme activity in met homozygotes [149]. The COMT enzyme is a key regulator of synaptic dopamine levels, particularly in the frontal cortex. In healthy subjects, the met/met genotype is associated with improved performance on working memory and attentional control tasks activating the prefrontal cortex [150]. In this way, the *COMT* Val158Met polymorphism is a good candidate for modulating the executive deficits in PD. In a cohort of patients with early PD, met alleles were associated with impaired performance on planning tasks dependent on prefrontal cortical areas [151]. This finding was replicated in the CamPaIGN cohort [152]. Interestingly executive performance improved in individuals over 5 years of disease progression, suggesting that despite its association with cognitive performance cross-sectionally, this gene is not necessarily associated with cognitive decline or with dementia risk. 

*BDNF* is widely distributed in cortical and subcortical areas, where it functions to maintain neuronal survival and promotes synaptic plasticity, dendritic morphogenesis, arborization and even neurogenesis in the adult brain. In the developing brain, *BDNF* is crucial for the proper establishment of dopaminergic neurons in the substantia nigra [153]. The G196A (Val66Met) polymorphism is one of the most frequently studied variants in the *BDNF* gene. Several studies have analyzed the effect of the Val66Met polymorphism on cognition in PD [154]. One study showed that PD patients that were BDNF Met carriers suffered cognitive impairment more frequently and had lower MMSE scores [155,156]. However, other studies have failed to support this association [156,157,158,159,160]. Thus, the role of BDNF in cognitive function in PD remains unclear. 

## 4. Protective Factors

### 4.1. Exercise

Exercise is another important lifestyle consideration. The general health benefits of exercise are well-known and there is reliable evidence for significant benefits on cognition in healthy older adults, especially for executive control processes [161]. Since executive dysfunction is a key finding in PD-MCI and PDD, exercise may be a relevant factor preventing or modifying cognitive impairment in PD. In the Parkinson’s Environment and Gene study, higher lifetime average physical activity was associated with less decline on the MMSE [138]. A small study showed that patients with PD who participated in a multimodal exercise program had statistically significant improvement in executive function, measured by the Wisconsin Card Sorting Test, compared to a group of patients who kept to their usual daily routine [162]. A randomized single-blind, pilot trial of patients with mild to moderate PD also showed that treadmill training was associated with significantly improved global executive function, compared to a control group [163]. Resistance exercise may also benefit cognition in PD. After 24 months of twice-weekly progressive resistance exercise, adults with PD improved on working memory, inhibition, and attention [164]. Another group used a combined aerobic and strength-training regimen, with cognitive improvement following exercise training [165]. The above suggest that exercise could be a feasible intervention to improve executive function in PD. Interestingly, some studies suggest that high intensity exercise may lead to an increase in reactive oxygen species, which theoretically could lead to increased brain oxidative stress. This effect has not been observed with moderate intensity exercise [166,167,168]. Most of these studies have been conducted in murine models and their generalizability to humans remains to be seen. Nonetheless, this underscores the importance of determining the frequency, intensity, type, and timing of exercise that may be most effective in PD. Additionally, the effect of exercise on preventing cognitive impairment has not been fully elucidated and has not, to our knowledge, been studied in randomized trials. Future randomized controls trials in larger cohorts, as well as in prodromal PD groups are needed. 

### 4.2. Diet

Diet is a complex lifestyle factor to study since it represents a combination of many individual food components as well as vitamin and mineral supplementation. Foods and other dietary components have received significant attention as potentially important environmental factors related to the pathogenesis of and protection against PD. There is also interest in describing which nutrients and food may impact the development of cognitive changes in PD, but the body of literature in this area is small. The Mediterranean diet has been of interest given its reported association with cognitive health in the general population and in other neurodegenerative disorders, such as AD [169,170]. In one study, patients with PD were randomly assigned to either an individually designed Mediterranean diet or usual dietary intake. After 10 weeks, those in the intervention group had significantly improved MoCA scores in the executive function, language, attention, concentration and active memory tasks, while they were unchanged in the control group. There was no significant change in visual-spatial ability, memory learning or navigation tasks [171]. The potential mechanisms regarding these effects are unknown, although a reduction in vascular risk factors, particularly hypertension and dyslipidemia [172], beneficial effect on oxidative stress and an anti-inflammatory effect may be playing a role. 

In some studies, 25-hydroxyvitamin D 25(OH)D has been found to be lower in PD patients compared to controls [173] and low vitamin D levels might contribute to the development of dementia in the general population [174]. However, the relationship between 25(OH)D and clinical features of PD is inconsistent. In one study, PD patients had significantly lower 25(OH)D and MMSE scores when compared to healthy controls. Among PD patients, those with vitamin D deficiency had a greater impairment of global cognitive function [175]. Another study following neuropsychiatric function in 286 PD patients found that higher 25(OH)D levels were associated with better semantic fluency and memory [176]. Nonetheless, a different prospective observational study compared newly diagnosed PD with age-matched controls; while 25(OH)D levels were lower in PD patients both at baseline and at 36 months, there was no association with cognitive measures [177]. More studies are needed to ascertain the role of 25(OH)D in cognitive changes in PD, as well as the potential role of 25-(OH)D supplementation for possible benefits in cognitive function. 

Several longitudinal studies support an inverse association between caffeine consumption and decreased memory impairment associated with aging, as well as reduced risk of dementia and AD [178,179]. Coffee has been inversely associated with PD risk [180]. A cross-sectional study involving treatment-naïve PD patients showed that coffee drinking was significantly associated with a reduced severity in the cognitive domain of the non-motor symptom assessment scale [181]. In the Parkinson’s Environment and Gene study PD patients also reported on their average caffeinated beverage consumption and ever coffee consumption was associated with less decline in MMSE [138]. In a cohort of newly diagnosed, treatment-naïve PD patients, there was an association between the number of cups of coffee per day and MMSE and MoCA scores, although statistical significance was lost after adjusting for confounders [182]. The Café-PD study was a multicenter parallel-group controlled trial where patients were randomized to caffeine or placebo. Interestingly, MoCA scores at 18 months were worse in the caffeine group; nonetheless, this was mostly accounted for by improvement in the placebo group, and this was an exploratory finding, which, due to the multiple comparisons in the study, may have been due to chance [183]. 

## 5. Future Directions

**Figure 1:**
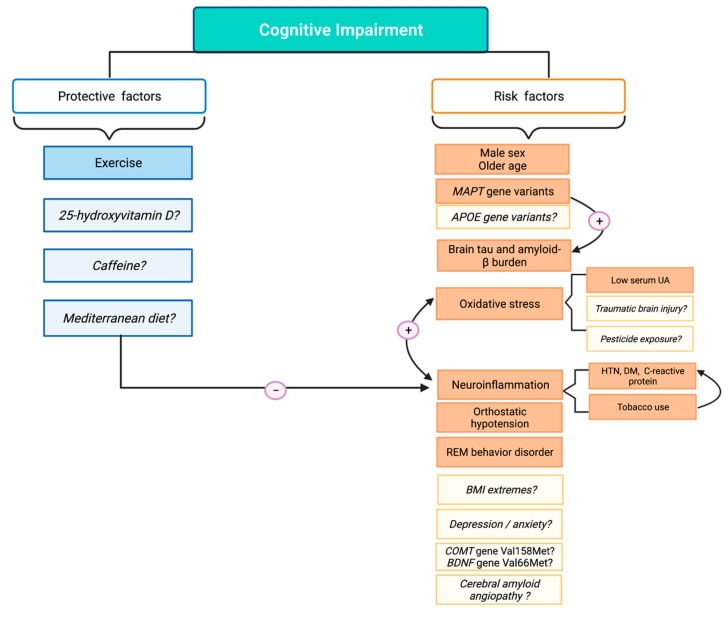
Protective and risk factors for cognitive impairment. Factors associated with cognitive impairment are classified as either protective or risk factors. Established factors in each category are shown in solid colors. Factors where the association remains uncertain are shown in light colors and emphasized with a question mark. Possible interactions and mechanisms of action are displayed by connecting arrows and brackets. APOE = Apolipoprotein E; BDNF = Brain-derived neurotrophic factor; BMI = body mass index; COMT = Catechol-O-methyltransferase; DM = diabetes mellitus; HTN = hypertension; MAPT = Microtubule-associated protein tau; REM = rapid eye movement; UA = uric acid. Created with BioRender.com.

Cognitive impairment has a major impact over the course of Parkinson’s disease and contributes substantially to disease burden, particularly as the disease progresses but even early in the disease. As such there is increasing interest in understanding the factors associated with the development of PD-MCI and PDD in order to develop preventive or modifying strategies. To date, there is conflicting evidence for most environmental and genetic factors that have been studied (Figure 1). Contributing methodological issues include the use of inconsistent criteria to define PD-MCI and PDD, the use of different instruments to measure cognition. In addition, cognition is a multifaceted function that is complex to measure, and many studies lack a comprehensive neuropsychological assessment. This hinders the description of specific cognitive patterns associated with specific risk factors. One additional issue is the timing of exposure; there may be an etiologically relevant time window where a given exposure may be causally related to disease onset. In this way, measurement of lifetime exposure to environmental factors can provide information related to cognitive decline onset, while exposure to the same factors once cognitive decline has started may give us information on how these contribute to disease course. The complication lies in identifying the correct time window, which has been historically difficult in PD given its long prodromal phase and long symptomatic course. 

Although the development of cognitive impairment has been well documented for some genetic forms of PD, such as *GBA*, the effect of other genetic variants and environmental exposures is much less clear. Regarding genetic variants, the role of less frequent variants, such as *SNCA* or *PINK1* variants is unclear, mainly due to the small number of cases reported. Importantly, most of the studies analyzing genetic risk factors have included mostly Caucasian subjects. As such, the role of these genes in other populations still needs to be ascertained. The same can be said for variants in genes such as *COMT, APOE, BDNF*. Our understanding of the role of environmental factors is similarly limited; while factors such as traumatic brain injury and pesticide exposure have been extensively studied as risk factors for the development of PD, the specific role they play in cognition, other aspects of the symptom complex of PD and PD progression needs to be better understood. 

Even less is known about protective factors. Cohort studies focused on prodromal PD may provide an opportunity to explore this, by prospectively documenting lifestyle, dietary and other environmental risk factors prior to the onset of or early in the course of cognitive decline. In particular, identification of modifiable risk factors, such as specific diet patterns, dietary supplementations or exercise regimens should be prioritized, as this will allow for potential interventions even in established PD. Evidence for a role of ongoing environmental exposures in accelerating or decelerating the progression of PD provide an important basis for targeted interventions or counselling to improve the lives of people living with PD. Adding further complexity, there is increasing awareness of interactions between gene and environment as determinants of disease phenotype, and this large field has been practically unexplored regarding cognition and PD. As genetic testing becomes more accessible, the elucidation of risk variants may allow for the identification of at-risk individuals who may benefit most from interventions.

## Figures and Tables

**Table 1:** Best performing tests to detect PD-MCI as reported by Goldman et al [32].

Attention/ Working Memory	Trail Making Test-Part A
	Symbol Digit Modalities Test
**Executive function**	Trail Making Test-Part B
	Clock Drawing Test
**Visuospatial functioning**	Judgment of Line Orientation
	Intersecting pentagons
**Language**	Boston Naming Test
	Animal naming
**Memory**	Free and Cued Selective Reminding Test
	Figural Memory

## Data Availability

Not applicable.
